# The Interplay Between Cardiovascular Disease and Lung Cancer

**DOI:** 10.7759/cureus.62953

**Published:** 2024-06-23

**Authors:** Luka Katic, James Choi, Sara Diaz Saravia, Alexander Silverman, Alexander Nagourney, Vincent Torelli, Soumya Gupta, Martina Glavan, Amit Gulati, Sakshi Khurana, Elina Tsyvkin

**Affiliations:** 1 Internal Medicine, Icahn School of Medicine at Mount Sinai, New York, USA; 2 Neuroscience, Yale University, New Haven, USA; 3 Cardiology, Icahn School of Medicine at Mount Sinai, New York, USA; 4 Radiology, New York Presbyterian-Columbia University Irving Medical Center, New York, USA; 5 Center for Hematologic Malignancies, Memorial Sloan Kettering Cancer Center, New York, USA

**Keywords:** multidisciplinary care, cancer treatment complications, shared risk factors, lung cancer, cardiovascular disease

## Abstract

Cardiovascular disease (CVD) and lung cancer are among the leading causes of mortality worldwide, with a significant interplay that complicates patient management and treatment outcomes. This review explores the complex relationship between various forms of CVD - such as coronary artery disease, heart failure (HF), arrhythmias, and valvular heart disease - and lung cancer. Shared risk factors, including smoking, aging, and chronic inflammation, contribute to the co-occurrence of these conditions. Additionally, treatments for lung cancer, particularly chemotherapy and radiation therapy, can exacerbate CVD, necessitating a multidisciplinary approach to patient care. We delve into specific CVD-related impacts on lung cancer prognosis and vice versa, examining mechanisms, clinical outcomes, and management strategies. Our findings highlight the need for integrated care involving oncologists, cardiologists, and other healthcare providers to optimize treatment plans and improve patient outcomes. Emphasizing comprehensive cardiovascular risk management in lung cancer patients, we advocate for further research to deepen our understanding and develop novel therapeutic approaches, ultimately enhancing the quality of life and survival rates in patients suffering from both CVD and lung cancer.

## Introduction and background

Cardiovascular disease (CVD) and lung cancer are two of the most significant health challenges globally, associated with high rates of morbidity and mortality. This review focuses on the complex relationship between CVD - which includes conditions like coronary artery disease, heart failure (HF), arrhythmias, valvular heart disease, and chronic cardiovascular risk factors like diabetes, hyperlipidemia, thyroid diseases, and hypertension - and lung cancer, the leading cause of cancer-related deaths worldwide. In recent years, the interconnection between these two conditions has gained more attention, revealing a complex interplay that affects how patients are managed and treated [1].

Globally, CVD is the number one cause of death, responsible for an estimated 17.9 million lives lost each year [2]. Diseases under the CVD umbrella, such as ischemic heart disease (IHD), cerebrovascular disease, and HF, have distinct characteristics and effects on health. On the other hand, lung cancer causes around 1.8 million deaths annually, marking it a critical public health issue [3]. Despite the declining trend in mortality due to advancements in prevention and treatment, the interconnection between CVD and lung cancer remains a concern [1]. This connection is multifaceted, with shared risk factors like smoking, aging, and inflammation playing a significant role [4]. Furthermore, treatments for lung cancer, including chemotherapy and radiation, can have harmful side effects on the heart, highlighting the need for careful management of these patients [5].

This review aims to shed light on how specific cardiovascular diseases interact with lung cancer, exploring the underlying mechanisms and their implications for treatment. By reviewing the latest research and clinical outcomes, we intend to offer insights into better management practices for patients afflicted with both cardiovascular disease and lung cancer. We believe our findings will contribute to the broader discourse on improving therapeutic approaches for this group of patients, emphasizing the need for a comprehensive understanding of their interplay.

## Review

Overview of cardiovascular disease and lung cancer

1.1. Cardiovascular Disease

The cardiovascular system is essential for sustaining life, distributing nutrients, and removing wastes through blood circulation. It can be analyzed through four primary functions: coronary, muscle, electrical, and valvular. These aspects offer a framework for understanding the complex nature of CVDs, which encompass a range of conditions affecting the heart and blood vessels [6]. Additionally, chronic conditions that accompany CVDs, like diabetes mellitus, hyperlipidemia, thyroid diseases, and hypertension play a big role in long-term cardiovascular survival [7].

1.1.1. Ischemic heart disease: IHD or coronary heart disease, involves inadequate blood supply to the heart muscle, primarily due to atherosclerosis in coronary arteries or, less frequently, microvascular disease. Patients can experience either chronic (stable) or acute (unstable) manifestations of the disease, with the majority diagnosed with chronic coronary syndrome (CCS) based on typical angina symptoms and risk factors. Diagnosing CCS may involve stress testing or anatomic assessments like coronary CT angiography, especially in cases with atypical symptoms or silent ischemia. The severity of coronary artery disease (CAD) is crucial for determining its treatment. The severity can be assessed with stress tests, imaging, and angiography to evaluate coronary obstruction and left ventricular (LV) function.

Treatment strategies range from medical management for low- to intermediate-risk patients to revascularization for high-risk cases or those with refractory angina. Revascularization options include percutaneous coronary intervention (PCI) and coronary artery bypass graft surgery (CABG). Antianginal therapy utilizes beta-blockers, calcium channel blockers, nitrates, and ranolazine to manage symptoms, with beta-blockers recommended as the first line of treatment. Prevention of disease progression emphasizes risk factor control, including antiplatelet therapy, statins, and lifestyle modifications such as exercise and stress reduction. Regular follow-up is necessary to monitor symptom changes, treatment tolerance, and risk factor management. Management considerations for older adults involve careful medication dosing and monitoring for side effects. The goals of CCS management are to relieve symptoms, secure diagnosis, evaluate disease extent, and prevent future cardiac events [8].

1.1.2. Heart failure: HF, characterized by inadequate blood pumping or high filling pressures, can result from various structural or functional cardiac disorders. Diagnostic tests, while crucial, cannot replace clinical judgment; elevated pulmonary capillary wedge pressure during rest or exercise in symptomatic patients is a definitive diagnostic indicator. HF can be triggered by LV dysfunction, right ventricular (RV) dysfunction, valvular heart disease, and other conditions, classified by ejection fraction (EF) into HF with reduced EF (HFrEF, EF ≤40%), mid-range EF (HFmrEF, EF 41-49%), and preserved EF (HFpEF, EF ≥50%). HF's functional status is often categorized using the New York Heart Association (NYHA) classification. Its symptoms include fluid accumulation signs (dyspnea, orthopnea, edema) and reduced cardiac output signs (fatigue, weakness). Clinical features like older age, hypertension, and a history of myocardial infarction increase the HF likelihood. Physical examination can reveal findings like elevated jugular venous pressure, displaced apical impulse, and signs of volume overload.

Initial tests for suspected HF include ECG, which is particularly revealing for HFrEF but less so for HFpEF; natriuretic peptide levels, aiding in HF presence assessment; and a chest radiograph to differentiate HF from pulmonary diseases. An echocardiogram assesses EF, cardiac structure, and function, crucial for diagnosing and identifying HF causes. B-type natriuretic peptide (BNP) or NT-proBNP levels help diagnose HF in uncertain cases, with values varying by age indicating HF likelihood. However, while interpreting natriuretic peptides, factors like obesity, renal failure, and sacubitril-valsartan treatment effects should be considered [9,10]. A hemodynamic exercise test, involving right heart catheterization to measure filling pressures at rest and during exercise, serves as a definitive HF diagnostic modality in ambiguous cases. Other tests, such as cardiopulmonary exercise testing, may clarify HF's role in symptoms and functional impairment [11]. HF diagnosis requires integrating clinical evaluation, symptoms, signs, and test results. When assessing preoperative risk, the patients in general fall into one of the following three categories:

- Low risk: patients with well-controlled HF (NYHA Class I or II) without recent exacerbations and with good functional capacity (able to meet >4 METs without symptoms).

- Intermediate risk: patients with moderate HF (NYHA Class II or III), controlled on medication, without recent decompensation but with reduced functional capacity.

- High risk: patients with severe HF (NYHA Class III or IV), recent decompensation, EF significantly reduced (especially <30%), high levels of natriuretic peptides, or those with significant comorbid conditions [12].

1.1.3. Arrhythmias: Arrhythmias, or irregular heartbeats, can manifest as tachycardia (a fast heartbeat over 100 beats per minute) or bradycardia (a slow heartbeat under 60 beats per minute). Symptoms vary widely; some individuals may experience no symptoms, while others may have palpitations, lightheadedness, fainting, shortness of breath, or chest pain. Serious arrhythmias can lead to stroke, HF, or sudden death. Arrhythmias are classified into four main categories: extra beats (premature atrial, ventricular, and junctional contractions), supraventricular tachycardia [atrial fibrillation (AF), atrial flutter, and paroxysmal supraventricular tachycardia], ventricular arrhythmias (ventricular fibrillation and ventricular tachycardia), and bradyarrhythmias (caused by sinus node dysfunction or atrioventricular conduction disturbances) [13]. The root cause of arrhythmias is typically related to issues in the heart's electrical conduction system [14]. Diagnosis often involves ECG and Holter monitoring.

Treatments range from medications (like beta-blockers and antiarrhythmic agents) to medical procedures (such as pacemaker insertion) and surgeries. In emergencies or severe cases, cardioversion or defibrillation may be used to restore a normal heart rhythm [15]. Arrhythmias are a global health concern, affecting millions of people. AF, one of the most common types, impacts about 2-3% of people in Europe and North America [16]. Arrhythmias are increasingly associated with age and can also occur in children [17]. They can lead to sudden cardiac death and are for half of all cardiovascular disease deaths and about 15% of all global deaths, with ventricular arrhythmias accounting for the majority of sudden cardiac death cases [18]. During the coronavirus disease 2019 (COVID-19) pandemic, arrhythmias have been associated with high morbidity and mortality among hospitalized patients due to myocardial injury caused by the infection [19]. The management of arrhythmias depends on the type and severity of the arrhythmia, ranging from physical maneuvers and medications to electrical treatments like cardioversion, defibrillation, and pacing. In some cases, catheter ablation can provide a cure by removing abnormal heart tissue causing the arrhythmia [20].

1.1.4. Valvular heart disease: Valvular heart disease involves dysfunction of one or more of the heart's valves, potentially affecting the aortic, mitral, pulmonic, or tricuspid valves. These conditions primarily result from aging, but congenital abnormalities, rheumatic heart disease, and pregnancy also play a role. The heart valves regulate blood flow through the heart and major vessels, and their failure can significantly impact heart functionality, potentially requiring medication, surgical repair, or valve replacement.

The diseases can be classified based on the valve affected and the nature of the dysfunction: stenosis (narrowing that restricts blood flow) or insufficiency/regurgitation (failure to close properly, allowing backflow). Left heart diseases (affecting the aortic and mitral valves) are more common due to higher pressures on the left side of the heart. Aortic stenosis is often caused by calcification or congenital bicuspid valves, limiting blood ejection from the left ventricle. Aortic insufficiency typically involves valve leaflets failing to close properly, possibly due to connective tissue or immune disorders. Mitral stenosis, mainly from rheumatic heart disease, is rare and involves obstruction of blood flow from the left atrium to the left ventricle. Mitral insufficiency often results from left heart dilation due to heart failure, affecting the mitral valve's operation. Pulmonary and tricuspid valve disorders are considered right heart diseases, with pulmonary valve diseases being the least common in adults. Pulmonary stenosis usually arises from congenital issues, while insufficiency is generally mild and often does not require intervention. Tricuspid stenosis is rare and usually results from rheumatic disease, while tricuspid insufficiency is more common and can be a result of right ventricle dilation.

Diagnosis of these conditions involves a combination of chest X-rays, ECG, and echocardiography, which can provide detailed information about the valve's appearance and function. Treatment may include lifestyle adjustments, medications to manage symptoms or prevent complications, and in severe cases, surgical interventions such as valve repair or replacement [21-22].

1.2. Lung Cancer

Lung cancer remains a major health concern worldwide and is the leading cause of cancer-related deaths. It primarily comes in two forms: small-cell lung cancer (SCLC) and non-small-cell lung cancer (NSCLC), with NSCLC making up about 85% of all cases. This category includes several types of lung cancer, such as adenocarcinoma, squamous cell carcinoma, and large cell carcinoma. On the other hand, SCLC is known for its aggressive behavior and quick spread [23]. Globally, lung cancer caused approximately 1.8 million deaths in 2020. Trends in mortality have shown a decrease, particularly due to a drop in smoking rates, the main risk factor for developing lung cancer. Despite this positive trend, lung cancer deaths remain high, emphasizing the need for effective treatment and prevention strategies [24-25].

In recent years, treatment for lung cancer has seen significant advancements, particularly with the emergence of targeted therapies and immunotherapies for NSCLC. These advancements are tailored to attack specific cancer cells based on genetic markers, such as mutations in the epidermal growth factor receptor (EGFR), anaplastic lymphoma kinase (ALK), and c-ros oncogene 1 (ROS1) genes. These treatments have provided great breakthroughs, offering better outcomes and fewer side effects compared to traditional chemotherapy [26]. Immunotherapy, which boosts the body's immune response against cancer cells, has also made a significant impact. Medications like nivolumab, pembrolizumab, and atezolizumab have become vital options for NSCLC, showing promise in improving survival rates for patients [27]. However, managing lung cancer remains complex due to issues like drug resistance, disease recurrence, and adverse effects of treatments. Particularly, the cardiovascular effects of lung cancer treatments pose challenges, highlighting the need for coordinated care involving oncologists, cardiologists, and other specialists. This collaboration is crucial to managing the intricate relationship between lung cancer and CVD effectively.

Mechanisms of the interplay between cardiovascular disease and lung cancer

2.1. Coronary Artery Disease and Lung Cancer

The relationship between CAD and lung cancer is intricate and significantly affects how patients are treated, as shown in Table 1. Among all cancers, lung cancer is most strongly linked to the development of IHD. In the lung cancer population, 67% of deaths related to CVD were due to IHD in 1999, a figure that decreased to 52% by 2019 [1]. Studies have shown that severe CAD can significantly increase the risk of lung cancer, especially in patients with high inflammation levels. This suggests that people with severe CAD should undergo thorough screenings for lung cancer [28,29]. On the other hand, when a population at risk is screened for lung cancer, the presence of coronary artery calcium on low-dose CT scans can help predict overall mortality and the likelihood of cardiovascular events [30].

**Table 1:** Association between major CVD categories and lung cancer CVD: cardiovascular disease

CVD category	Estimated association with lung cancer
Coronary artery disease	High association: lung cancer patients have a significantly increased risk of coronary artery disease due to shared risk factors and the cardiotoxic effects of cancer treatments
Pump function (heart failure)	High association: lung cancer treatments, especially those involving radiation and certain chemotherapies, can exacerbate or lead to the development of heart failure
Valvular heart disease	Moderate to high association: radiation therapy for lung cancer can lead to valvular heart disease, with a higher risk for patients undergoing thoracic radiation
Electrical function	Moderate association: treatments for lung cancer, including chemotherapy and targeted therapies, can induce arrhythmias, although the direct link between lung cancer and arrhythmias varies

Patients with NSCLC who also have a history of CAD are at a higher risk of experiencing complications after lung surgery. While CAD does not seem to affect the long-term outcomes after surgery, it does make the recovery period riskier due to the combined effect of reduced organ function, a known factor for complications [31]. When treating both CAD and lung cancer, if a patient has a CAD lesion causing symptoms, it is usually addressed before any lung surgery. In some cases, it is possible to perform heart surgery and lung surgery at the same time, which can work well for patients with early-stage lung cancer. However, in higher-risk situations, it might be safer to perform the surgeries in stages, starting with the heart surgery and then doing the lung surgery a few weeks later [32].

On the flip side, lung cancer itself can be a risk factor for developing CAD, through shared risk factors like inflammation and smoking [33], or due to additional risks posed by the cancer, such as increased blood clotting. Treatments for lung cancer, including chemotherapy and radiation therapy, can also contribute to the risk of CAD, particularly for patients with tumors on the left side of the lung [34,35]. Also, treatments like the immunotherapy drug atezolizumab, used for NSCLC, have been linked to CAD, even though the primary risk with immunotherapy is myopericarditis. Autopsies of patients who passed away after receiving atezolizumab have shown instances of coronary artery tumor embolization [36]. On another note, certain tyrosine kinase inhibitors (TKIs), such as the EGFR inhibitor osimertinib, are known for their cardiotoxic effects but do not seem to significantly increase the risk of CAD [37]. However, EGFR inhibitors like erlotinib and gefitinib have been reported to lead to coronary artery events [38].

2.2. Heart Failure and Lung Cancer

Heart failure, as part of CVD mortality linked to lung cancer, increased from 3.6% in 1999 to 5.9% in 2019 [1]. Although there does not seem to be a direct link between heart failure and the development of lung cancer, heart failure does pose a risk for patients with newly diagnosed lung cancer, especially when considering surgery. Complications after lung cancer surgery can include cardiac failure [39]. Additionally, lung cancer treatments such as radiotherapy can cause radiation-induced heart disease (RIHD), damaging the heart's coronaries, valves, and pericardium, which may lead to heart failure [40]. However, there is evidence showing that targeted cardiac radiotherapy can alleviate life-threatening heart failure in cases of advanced SCLC with cardiac metastasis [41].

Cancer treatments can lead to cancer therapy-related cardiovascular toxicity, which is a pivotal cause of heart failure in patients undergoing cancer treatment. EGFR inhibitors like afatinib and osimertinib have been linked to heart failure. There is also a risk of myocarditis during anti-PD1/anti-PDL1 therapy, which needs to be carefully distinguished from acute coronary syndrome and heart failure [38]. In one reported case, a patient with NSCLC experienced severe, but reversible, cardiac failure after treatment with bortezomib combined with chemotherapy [42].

2.3. Valvular Function and Lung Cancer

Recent studies have demonstrated the feasibility of detecting and measuring cardiac valve calcifications using low-dose, non-enhanced, non-triggered CT scans originally intended for lung cancer screening [43]. Although aortic valve calcifications can indicate a higher risk of future cardiovascular events, their prognostic value - beyond factors like age, smoking history, and the presence of coronary and aorta calcium - does not significantly aid in the short-term prediction of these events [44].

Radiation therapy for lung cancer raises concerns about RIHD, which affects various parts of the heart, including the coronary arteries, myocardium, heart valves, and the pericardial sac [45,46]. It is crucial to be aware that conditions such as severe rheumatic mitral valve stenosis (MVS) and aortic regurgitation might mimic lung cancer symptoms, with perihilar masses and mediastinal lymphadenopathy sometimes being solely the result of hemodynamic changes and focal lymphedema. This resemblance can lead to unnecessary stress and delay in surgery for patients with MVS, as they undergo extensive testing for suspected lung cancer [47].

Furthermore, heart valve disease is not just a concern for lung cancer patients. Radiation therapy aimed at the mediastinum, crucial for treating various thoracic cancers like Hodgkin lymphoma and breast cancer, is linked to an increased risk of radiation-induced heart valve damage, which manifests as valve fibrosis and calcification. This risk becomes clinically significant 10-20 years after exposure and is dependent on the radiation dose, time since exposure, and whether chemotherapy was also used [48].

2.4. Electrical Function and Lung Cancer

Arrhythmias, including supraventricular and ventricular types, atrioventricular blocks, and sinus bradycardia, are less common yet notable side effects of cancer treatments [49]. In lung cancer, the potential causes of arrhythmias range from alterations in ion channel membrane activity and calcium balance in heart cells, affecting their electrical properties. This can be triggered by various treatments like anthracyclines, taxanes, antimetabolites, and alkylating agents. TKIs also play a role by affecting ion channels and cell signaling pathways [50]. Immunotherapy can lead to arrhythmias, primarily through drug-induced myocarditis, although such cases are rare [51].

Different chemotherapy drugs, targeted therapies, and immunotherapies used in lung cancer treatment are associated with various cardiac arrhythmias. Drugs like doxorubicin can cause a broad spectrum of arrhythmias, including serious conditions like ventricular arrhythmias and AV block. Similar arrhythmic risks are seen with methotrexate and cyclophosphamide, which can lead to sinus bradycardia and supraventricular tachycardia. Other medications, such as vincristine and platinum compounds like cisplatin, have been linked to AF and other arrhythmias. Taxanes and various TKIs, including alectinib and osimertinib, are associated with sinus bradycardia and changes in heart rhythm, with osimertinib specifically linked with ventricular tachycardia. Immune checkpoint inhibitors, such as pembrolizumab and atezolizumab, can lead to a range of arrhythmias, from sinus tachycardia to complete AV block, and, in rare cases, sudden cardiac death [52].

There is growing evidence on the link between cancer and AF, though the exact relationship and mechanisms are not fully understood. Inflammation related to cancer, treatments, and other comorbidities might contribute to changes in the heart's atrial structure, increasing the risk of AF in cancer patients [53]. Early treatment with metoprolol or losartan post-lung cancer surgery has been shown to significantly reduce postoperative AF in patients with high levels of NT-proBNP, a heart stress marker [54]. ALK inhibitors, another class of drugs, have been associated with electrical disturbances in the heart [38]. Interestingly, no significant differences were observed in the need for appropriate implantable cardioverter-defibrillator (ICD) interventions between cancer patients and non-cancer patients, regardless of the reason for the ICD [55].

Chronic diseases and lung cancer

3.1. Diabetes and Lung Cancer

Before lung adenocarcinoma surgery, having a preoperative HbA1c level of 8.0 or higher is linked to a worse prognosis due to the risk of distant metastasis. It is crucial to monitor these patients closely after their surgery [56]. For those undergoing first-line immunotherapy, a higher HbA1c level (6.5% or above) is associated with poorer overall survival [57]. Studies have found that levels of HbA1c, C-peptide, and insulin-like growth factor-1 (IGF-1) are significantly higher in lung cancer patients than in healthy individuals. Specifically, C-peptide and IGF-1 levels are elevated in patients with SCLC and advanced stages of the disease, hinting at a connection with more aggressive cancer forms. Furthermore, lung cancer patients with diabetes mellitus also exhibit increased levels of these markers, suggesting a complex relationship between diabetes and lung cancer risk. This indicates that high blood sugar and related markers could contribute to the development and progression of lung cancer, potentially through effects on the immune system and hormone secretion by tumors. Shared risk factors between diabetes and lung cancer, such as lifestyle and environmental factors, have also been highlighted [58].

The level of HbA1c before treatment is an important prognostic indicator for locoregional recurrence-free survival in stage III NSCLC patients undergoing radical radiotherapy. Monitoring HbA1c levels before treatment and managing blood sugar aggressively might help prevent locoregional recurrence in these patients [59]. Interestingly, diabetic patients on metformin therapy have a reduced risk of lung cancer [60], while insulin-treated diabetic women have a 27% increased risk of developing lung cancer compared to those not treated for diabetes. This risk is especially significant in non-smoking women, suggesting that insulin treatment for diabetes may independently increase the risk of lung cancer. Insulin-treated diabetes is more likely to be associated with NSCLC and well-differentiated tumors, particularly in those who are former or current smokers [61].

Metformin is linked to improved survival among stage IV NSCLC patients with diabetes, suggesting a possible anti-cancer effect [62]. Moreover, metformin might boost the effectiveness of EGFR-TKI therapy and survival rates in type 2 diabetes patients with lung cancer [63]. Lastly, there has been a rare case of a 34-year-old woman with metastatic NSCLC developing new-onset autoimmune diabetes mellitus after nivolumab treatment, an occurrence seen in less than 1% of patients treated with immune checkpoint inhibitors. Despite no previous or family history of diabetes, she developed diabetic ketoacidosis following treatment, requiring continuous insulin infusion [64]. Clearly, there is a link between diabetes, its treatment, and lung cancer, as shown in Table 2, underscoring the need for careful management and monitoring of diabetic patients undergoing lung cancer treatment.

**Table 2:** Association between chronic diseases and lung cancer HbA1C: glycated hemoglobin; IGF-1: insulin-like growth factor-1; TTF-1: thyroid transcription factor-1

Chronic disease	Estimated association with lung cancer	Reason for association
Diabetes	Moderate to high	Elevated levels of HbA1c, C-peptide, and IGF-1 in lung cancer patients suggest a link with cancer progression and aggressiveness. Diabetes may influence lung cancer development through systemic inflammation and hormonal imbalances
Hyperlipidemia	Moderate	While lipid metabolism plays a role in cancer cell growth, the direct impact of hyperlipidemia on lung cancer risk and progression is less clear. The use of certain lung cancer treatments can lead to lipid metabolism disorders
Thyroid problems	Moderate	Changes in thyroid hormone metabolism and the presence of TTF-1 have implications for lung cancer prognosis. However, direct causation and the overall impact of thyroid disorders on lung cancer risk are complex and not fully understood
Hypertension	Low	The association between hypertension and lung cancer risk is less direct, with limited evidence suggesting a slight increase in risk among smokers. High blood pressure may contribute to a pro-inflammatory state that influences cancer development

3.2. Hyperlipidemia and Lung Cancer

In patients receiving lorlatinib for lung cancer, there is a notable risk of developing hyperlipidemia. This drug, a ROS1/ALK inhibitor, is specifically linked to significant increases in lipid levels [65]. Apart from lorlatinib, repotrectinib, another ALK inhibitor that crosses into the brain, has been connected to weight gain [66]. The reason behind the weight gain, as well as increased levels of triglycerides and cholesterol seen with these brain-penetrating ALK inhibitors, is believed to involve interactions with the hypothalamus [67-69]. Beyond affecting metabolism, ALK inhibitors are known to cause a variety of side effects including pain, vision changes, pigmentation issues, impacts on central nervous system (CNS) functions, and reproductive effects [69].

Research has also shown that many types of tumors, including those in lung cancer, exhibit increased expression of low-density lipoprotein (LDL) receptors and a higher uptake of LDL, indicating a link between lipid metabolism and tumor growth [70]. However, patients with advanced, inoperable lung cancer often experience hypocholesterolemia, likely related to cachexia and the body's increased catabolism during advanced disease stages [71]. Additionally, oxidative stress observed in lung cancer patients can lead to changes in serum markers associated with lipid metabolism [72]. This body of evidence underscores the complex relationship between lung cancer treatments, particularly ALK inhibitors, and changes in lipid metabolism, highlighting the need for careful monitoring and management of these side effects in patients.

3.3. Thyroid Problems and Lung Cancer

Research indicates that lung cancer can impact thyroid hormone metabolism, as is especially reflected in the reduced conversion of T4 to T3, which is evident from altered T4/T3 ratios. This effect is particularly noted in patients with types of lung cancer such as anaplastic small and large cell cancers, and it could potentially influence patient prognosis [73]. Additionally, the presence of thyroid transcription factor-1 (TTF-1) has been identified as a positive prognostic indicator for survival in patients with NSCLC, especially those diagnosed with adenocarcinoma [74]. TTF-1 is involved in several aspects of lung cancer, including genetic changes, tumor growth, metabolism, and the secretion of certain proteins. Its role in both promoting and inhibiting cancer, along with its participation in key biological processes, positions TTF-1 as an important element in the study of lung cancer and as a possible focus for treatments [75].

Although rare, instances of primary lung adenocarcinoma spreading to the thyroid gland have been described. Such cases can be identified through biopsies, for example, by looking for EGFR positivity [76]. Thyroid issues are common in NSCLC patients being treated with pembrolizumab, often marked by an early phase of hyperthyroidism, a significant presence of anti-thyroid antibodies, and potentially leading to better outcomes. This pattern of thyroid dysfunction is closely associated with the body's immune response [77].

3.4. Hypertension and Lung Cancer

Hypertension has not been found to directly increase the risk of dying from lung cancer. However, among current smokers, having high blood pressure slightly raises the risk of lung cancer death [78]. When looking at the connection between hypertension and cancer in different organs, the kidney is most often linked to pre-existing high blood pressure. However, studies have also found associations between high blood pressure and cancers of the colon and lung [79]. Interestingly, hypertension does not seem to affect the risk of developing brain metastases in patients with primary SCLC [80]. The proportion of cardiovascular deaths attributed to hypertension increased from 4.5% in 1999 to 11.7% in 2019. This significant rise might reflect several factors, including better cancer survival rates; an aging population; and the impact of cancer treatments, such as EGFR inhibitors, which can cause high blood pressure, on cardiovascular health [1]. There are numerous reasons for early vascular aging [81] and arterial stiffness can increase mortality in these patients [82]. Among these reasons, anthracyclines, alkylating agents, and TKIs are known to impact the elasticity of arterial walls. The changes brought about by these substances can be permanent and might manifest even after the cessation of treatment [83].

## Conclusions

This review examined the complex relationship between CVD and lung cancer, two major health issues globally. It focused on how these conditions intersect, highlighting shared risk factors such as smoking, aging, and inflammation, and the impact of cancer treatments on cardiovascular health. Specifically, we discussed the effects of certain CVDs on lung cancer outcomes and vice versa, providing a detailed overview of the current knowledge about this field. The findings emphasize the need for careful cardiovascular risk management in lung cancer patients, given the negative effects of cancer treatments on heart health. This relationship between CVD and lung cancer underscores the importance of a comprehensive care approach, necessitating collaboration among oncologists, cardiologists, and other healthcare providers to improve treatment plans and patient outcomes.

Future research should delve deeper into understanding the connections between CVD and lung cancer. This could lead to new treatment methods tackling both issues, thereby improving the quality of life and survival rates for patients. We also point to the need for greater awareness and proactive cardiovascular care in lung cancer patients, stressing the importance of prevention and early detection in mitigating the impact of these diseases. To sum up, the intricate link between CVD and lung cancer poses challenges but also creates opportunities for improving patient care. By promoting research and collaboration across disciplines, we can find better ways to treat these conditions, aiming for improved outcomes for patients suffering from both CVD and lung cancer.
